# Morphometric Characterization of Levator Ani Subdivisions in Healthy Controls and Patients: An MRI Study Using 3D PICS

**DOI:** 10.1007/s00192-025-06082-5

**Published:** 2025-03-20

**Authors:** Nina Jessica Gmür, Soleen Ghafoor, Klaus Steigmiller, Thomas Winklehner, Cäcilia S. Reiner, Cornelia Betschart

**Affiliations:** 1 Department of Gynecology, University Hospital Zurich, University of Zurich, Zurich, Switzerland; 2 Institute of Diagnostic and Interventional Radiology, University Hospital Zurich, University of Zurich, Zurich, Switzerland; 3 Department of Biostatistics, Epidemiology, Biostatistics and Prevention Institute, University of Zurich, Zurich, Switzerland; 4 ARTORG, University of Bern, Bern, Switzerland; 5 Department of Obstetrics and Gynecology, Medical University of Vienna, Vienna, Austria

**Keywords:** 3D pelvic inclination correction system (3D PICS), Insertion, Morphometry, MRI, Pelvic floor muscles, Pelvic organ prolapse

## Abstract

**Introduction and Hypothesis:**

To date, levator ani muscle (LAM) morphometry has been classified descriptively and semi-quantitatively. New MRI techniques enabling detailed visualization with the 3D pelvic inclination correction system (3D PICS) could offer a one-stop-shop diagnostic modality for quantitative assessment of LAM subdivisions. The aim of this controlled MRI study was to assess morphometric LAM subdivision characteristics in two distinct groups of premenopausal women, namely nulliparous asymptomatic controls and symptomatic patients (Pelvic Organ Prolapse Quantification [POP-Q] ≥ II).

**Methods:**

Magnetic resonance imaging scans of the 22 women in each group were analyzed applying the 3D PICS coordinate system. A second reading of MRI was used to calculate interrater reliability (IRR). Origins and insertions were expressed in the 3D-Cartesian coordinate system in relation to point 0/0/0 (inferior pubic point). Distances and angles between muscles and planes were described using mean and standard deviation or median with first and third quartiles for all LAM subdivisions.

**Results:**

Moderate to good IRR was reported except for points close to point 0/0/0. Origins showed no difference between groups. Insertions differed notably in the vertically oriented pubovaginal, puboperineal, and puboanal muscles, with patients exhibiting lower positions along the superior–inferior axis by 6.1–7.7, 8.8, and 8.0–8.2 mm respectively. In contrast, the insertions of the horizontally oriented puborectal muscle showed a smaller difference of 1.8 mm. Muscle lengths were also 4% to 24% longer in cases.

**Conclusions:**

This in vivo MRI study reveals first geometric 3D data on LAM morphology in 3D PICS for both cases and controls. Exact 3D coordinates of origin/insertion points, lengths, and angles could serve as a basis for future imaging-based POP diagnostics.

**Supplementary Information:**

The online version contains supplementary material available at 10.1007/s00192-025-06082-5

## Introduction

Levator ani muscle (LAM) disruption after first vaginal birth is found in 15–20% of women, with a mean latency between delivery and onset of pelvic organ prolapse (POP) symptoms of 33.5 years [1–3]. POP symptoms arise after one vaginal delivery [4]. The economic burden, estimated to be US$1.2 billion annually in the USA, is substantial and can be expected to increase with an ageing population [5].

In clinical practice, POP is usually classified descriptively and (semi-)quantitatively by gynecological examination or ultrasound imaging [6]. MRI can provide an early, non-invasive diagnostic approach to reproducible objective quantification. In a first detailed MRI-based description in 2003, the pubovisceral muscle was identified as the main focus of birth-related LAM injuries [1]. Since then, the LAM subdivisions have been shown in origin-insertion pairs and compared with their vectors of action [7, 8]. An MRI grading system with good interrater reliability and an ultrasound grading system that takes account of the defects seen on the MRI were also introduced [9–11]. In a 2013 comparison of ultrasound and MRI, agreement was overall good but lowest for the highest-grade defects [12]. Semi-quantitative measures of LAM structures distinguish between “normal” and “abnormal” muscle attachment in different patient groups and categorize the “abnormal” muscle appearance into either partial or complete avulsion [13]. Other quantitative measures are muscle volume cross-sectional areas as surrogate markers of muscle integrity [14].

However, as POP is a spatial condition, it is not precisely reflected by the previously introduced measurement systems with their semiquantitative descriptions.

The 3D pelvic inclination correction system (PICS) [15], expressing location precisely as a 3D coordinate combined with a tailored MRI protocol, has the potential to offer a one-stop-shop diagnostic modality, particularly for accurately identifying the site of LAM failure in the preoperative setting in the spatial analysis of LAM subdivisions (pubovisceral [PVM], pubovaginal [PVaM], puboperineal [PPM], puboanal [PAM], puborectal [PRM], iliococcygeus [ICM], and coccygeus [COC] muscles), thereby advancing pelvic floor diagnostics in various patient groups.

In a recent pilot and feasibility study by Moser et al., the LAM morphometry of nulliparous women was described for the first time, using 3D PICS [16]. The aim of this prospective controlled quantitative MRI study was to assess the 3D morphometrics of LAM subdivisions in two distinct premenopausal groups, nulliparous controls and symptomatic, parous cases (Pelvic Organ Prolapse Quantification [POP-Q] ≥ II), by applying 3D PICS.

## Materials and Methods

### Patients and Volunteers

Magnetic resonance imaging scans of 22 women from each of the two groups were included in the prospective study, which had been approved by the local ethics committee (Study ID: BASEC 2018–01107). Twenty-two consecutive volunteers and 22 parous patients from the urogynecology unit of our hospital were examined after giving their written informed consent to participate.

The healthy nulliparous volunteers were recruited by advertisements on hospital notice boards, whereas potential POP cases were recruited in the urogynecological department. Cases were required to have given birth vaginally and have symptomatic POP-Q ≥ II in the anterior or central compartment, which had been diagnosed in a gynecological examination according to the POP-Q value system [17]. All cases presented with POP of at least POP-Q stage II, with the most prominent point in the anterior or apical compartment measuring ≥ 0 (e.g., 0, + 1, + 2). Nulliparous, asymptomatic controls did not have to undergo a vaginal examination.

Both groups of participants filled out basic demographic information and the German-language pelvic floor questionnaire (PFQ) with the domains urinary incontinence, bowel function, sexual function and symptoms of descent. All had a PFQ total score ranging from 0 to 40 [18].

### MRI Protocol

The MRI, recorded between November 2019 and October 2021, consists of sagittal, axial, and transverse sequences of the whole pelvis. Informed consent was given.

The pelvic floor MRI was performed on a 3.0 T MRI system (Skyra; Siemens Healthineers, Erlangen, Germany) with a 60-channel array coil while the participant was in supine body position with an empty bladder. Static proton-density-weighted images were acquired in transverse, sagittal, and coronal planes (turbo spin echo sequence, TR/TE, 8200/9.6 ms; voxel size, 0.75 × 0.75 × 3 mm^3^; matrix, 320 × 320; FOV, 240 × 240 mm; slice thickness, 3 mm).

### Quantitative Image Analysis

The 3D PICS coordinate system developed by Reiner et al. in 2017 serves as a tool for the millimeter-precise determination of 3D spatial coordinates, such as the LAM origin and insertion points in relation to the PICS planes [15]. Anatomical bony landmarks (inferior pubic point and sacrococcygeal articulation in the midsagittal plane and bilateral ischial spines in the axial plane) are used as constant bony reference points within individual MRI datasets. The x-axis is the anterior–posterior axis, the y-axis the superior–inferior axis, and the z-axis the left–right axis.

The resulting PICS plane exhibits an alignment with respect to the x–z plane, forming a 90° angle in relation to the perpendicular body axis represented by the y-axis. The PICS plane in an upright person is individually defined and depends on her pelvic tilt. For a population average, it is a nearly horizontal plane [19]. This orientation is established to align with the gravitational force vector to mimic the physiological effects of prolapse in an upright position and allows for the computation of angular measurements within the pelvic region.

Origin and insertion locations (middle-distance point for fan-like muscles) are expressed as x/y/z coordinates in relation to point 0/0/0 at the inferior pubic point. Muscles with a clearly visible origin and insertion are the PRM and the ICM. The origin of the PVM is fan like, then splits downward into its insertions PVaM, PPM, and PAM.

Images were annotated according to the recently published instructions on muscle annotations [16] by a 2nd-year resident (N.G., basic experience with pelvic MRI). A detailed instruction with example images can be found in the supplementary material. For evaluation of the interreader agreement, a randomly chosen subset of 5 controls and 5 cases was annotated by a second reader (S.G., abdominal radiologist with 10 years of experience).

Distances and standard deviations (SD) in the 3D space for ICM and PRM were calculated using the Euclidean distance (Appendix 2). To calculate the distances for the PVM and its subdivisions, three artificial auxiliary points were introduced and defined as the first, second, and third equidistant points between the first and last origin points of the PVM (see Fig. 2). Angles of incidence were computed with reference to the transverse, coronal, and sagittal planes of the PICS system. We adopted the method for the normal vectors from our feasibility study [16]: “Directional vectors of the muscles were calculated by subtracting the origin coordinates from the insertion coordinates. Then, the normal vector of the coronal, transverse, and sagittal PICS planes were defined as the posterior, inferior and right normalized direction vector, respectively. The resulting angles, ranging from −90° to 90°, were reported. Muscles with a directional vector to the posterior, inferior and right were assigned a positive angle, while those pointing opposite were assigned negative angles.”

For muscles with three or more points, not all of the initially marked points were used in the analysis. For the PAM insertion, only the middle point was used, for the PVM origin and ICM origin only the two border points were used.

**Fig. 1:**
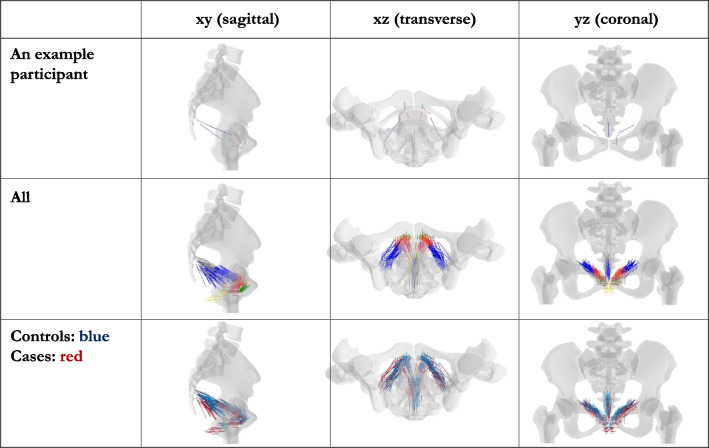
Visualizations: pubovisceral muscle origin in *bright red*, pubovaginal muscle insertion in *dark red*, puboperineal muscle insertion in *orange*, puboanal muscle insertion in *yellow*, puborectal muscle origin and insertion in *green*, iliococcygeus muscle origin and insertion in *blue*, coccygeus muscle insertion in *gray*

The software MATLAB was used to create a colored 3D visualization of the origin and insertion points for the different LAM subdivisions of the two collectives (Fig. 1). A supplementary 3D visualization was created as a video clip.

### Statistical Analysis

For descriptive analyses, mean and SD or median with first and third quartiles as well as frequency and percentage for nominal variables were reported. The coordinates of muscle origins, insertions, and bony landmarks as well as muscle lengths and angles between muscles and planes, were analyzed with means and SDs for all LAM subdivisions; for distances and lengths, medians with first and third quartiles were also calculated.

No sample size calculation was carried out as no comparable study exists to use as an exact model. The study collective was derived from a previous study that included pressure studies [20]. For this muscle comparison study the static MRI from the two collectives was utilized.

Intraclass correlation (ICC) was calculated based on the coordinates of 5 randomly selected cases and 5 controls by two different raters (N.G. and S.G.) and interpreted according to the Landis–Koch criteria (< 0.2: poor, 0.21–0.4: fair, 0.41–0.7: moderate, 0.71–0.80: substantial, and 0.81–1: almost perfect) [21]. In detail, all ICC estimates with corresponding 95% confidence intervals were calculated using the R package psych v2.1.9 [22]. All analyses were performed using R version 4.1.2; R Markdown was used for dynamic reporting [23, 24].

No missing values were recorded for the morphometric and reliability analyses; therefore, no methods for missing values were required.

## Results

### Demographics

In total, 48 women were recruited. One case was excluded owing to a lack of POP and 3 women were lost as MRI recording was not possible, yielding 44 women eligible for analysis.

Demographics were comparable except for higher age in POP cases. In the case group, the median for vaginal deliveries was 2, 13% of which were instrumental, with a mean fetal weight of 3684 ± 540 g. Mean PFQ score was 10.05 ± 5.5, with 10 women showing POP-Q II and 12, POP-Q III. Six women in the case group did not fill out the PFQ subsection “sexual function” owing to sexual inactivity. Women in the control group were nulliparous, with a mean PFQ score of 1.74 ± 1.35 (Table 1).

**Table 1:** Participant characteristics, delivery mode, pelvic floor questionnaire (PFQ), Pelvic Organ Prolapse Quantification (POP-Q)

Characteristic	Control group	Case group
Age (years) mean ± SD	23.7 ± 3.8	38.6 ± 4.3
Height, (m) mean ± SD	1.66 ± 0.1	1.68 ± 0.1
Weight (kg) mean ± SD	60.5 ± 10.1	63.2 ± 12.2
BMI (kg/m^2^) mean ± SD	21.9 ± 3.2	22.5 ± 3.8
Ethnicity	21 white, 1 Asian	22 white
Delivery mode, median (range)		
Spontaneous		2 (0–4)
Vacuum/forceps		0 (0–1)
Caesarean		0 (0–1)
Fetal weight (g) mean ± SD		3684 ± 540
Last delivery to MRI recording (months) median (1st quartile and 3rd quartile)		25 (10–142)
PFQ		
PFQ score total, mean ± SD (*n* = 41)	1.74 ± 1.35	10.05 ± 5.5
PFQ score UI, mean ± SD (*n* = 41)	0.41 ± 0.48	1.99 ± 1.42
PFQ score BF, mean ± SD (*n* = 41)	1.04 ± 0.72	2.31 ± 1.67
PFQ score POP, mean ± SD (*n* = 41)	0.0 ± 0.0	3.94 ± 2.49
PFQ score sex, mean ± SD (*n* = 35)	0.42 ± 0.74	1.7 ± 1.78
POP-Q		10 II, 12 III
Aa, mean ± SD		−0.14 ± 1.2
Ba, mean ± SD		0 ± 1.3
C, mean ± SD		−1.64 ± 3.1
D, mean ± SD		−3.64 ± 2.3
Ap, mean ± SD		−1.23 ± 1.2
Bp, mean ± SD		−0.91 ± 1.5
Tvl, mean ± SD		9.68 ± 1.0
Gh, mean ± SD		5.59 ± 0.8
Pb, mean ± SD		3.09 ± 0.5

*UI* urinary incontinence, *BF* bowel function, *POP* pelvic organ prolapse, *sex* sexual function

### 3D Organ and Bony Landmark Location

The bladder, cervix, and anorectum all lie higher in controls than in cases (y-axis; Table 2; Appendix 1). The cervix showed the highest variability, with 36 ± 12.5/−55.2 ± 7.6/−8.2 ± 10.4 mm in the x-/y-/z-axis in controls and 37.9 ± 11.7/−45.0 ± 15.8/0.1 ± 10.0 mm in cases. The bony landmarks show similar locations with an SD of 4–7 mm.

**Table 2:** Left-sided analysis of the marked 3D PICS muscle, organ, and bone points (x/y/z) on each axis; mean values are expressed in mm (± SD)

Point	Controls			Cases		
	x	y	z	x	y	z
Bladder	−0.7 ± 5.3	−26.5 ± 2.1	−1.4 ± 3.5	7.4 ± 8.2	−18.5 ± 9.4	1.4 ± 3.9
Cervix	36.4 ± 12.5	−55.2 ± 7.6	−8.2 ± 10.4	37.9 ± 11.7	−45.0 ± 15.8	0.1 ± 10.0
Anorectum	27.7 ± 4.5	−23.5 ± 6.8	−1.1 ± 3.2	35.7 ± 6.9	−15.9 ± 8.5	0.7 ± 4.5
Symphysis	0.0 ± 0.0	0.0 ± 0.0	0.0 ± 0.0	0.0 ± 0.0	0.0 ± 0.0	0.0 ± 0.0
Ischial spine	55.5 ± 6.5	−47.9 ± 3.8	−57.5 ± 5.4	51.9 ± 4.2	−47.0 ± 4.7	−56.3 ± 4.4
Coccyx	91.7 ± 6.3	−61.8 ± 4.3	0.0 ± 0.0	92.2 ± 5.8	−62.2 ± 3.9	0.0 ± 0.0
PVM origin 1	9.3 ± 4.3	−34.5 ± 4.3	−30.3 ± 4.2	13.7 ± 4.4	−29.4 ± 5.4	−31.2 ± 4.5
PVM origin 3	−4.3 ± 3.5	−14.0 ± 3.1	−16.6 ± 4.8	0.8 ± 5.4	−12.2 ± 3.8	−19.0 ± 6.4
PVaM insertion 1	5.3 ± 3.0	−12.3 ± 3.9	−16.2 ± 3.5	11.3 ± 5.0	−4.6 ± 6.6	−13.4 ± 5.9
PVaM insertion 2	17.4 ± 3.1	−11.6 ± 3.7	−12.5 ± 3.6	25.3 ± 5.7	−5.2 ± 5.6	−10.4 ± 5.5
PPM insertion	22.0 ± 5.0	−3.6 ± 5.6	−1.9 ± 2.8	36.6 ± 8.6	5.2 ± 7.2	1.1 ± 3.0
PAM insertion	38.3 ± 6.1	1.5 ± 6.8	−9.0 ± 2.7	52.6 ± 9.8	9.7 ± 7.2	−6.8 ± 3.0
PRM origin 1	−8.5 ± 3.5	−14.4 ± 3.2	−16.9 ± 4.4	−7.0 ± 2.6	−12.4 ± 4.4	−15.7 ± 5.5
PRM origin 2	2.5 ± 2.1	−2.8 ± 1.6	−17.0 ± 4.4	2.7 ± 1.9	−1.8 ± 3.1	−15.7 ± 5.5
PRM insertion 1	40.3 ± 6.1	−6.5 ± 5.1	−1.4 ± 2.5	51.7 ± 8.7	−4.7 ± 7.8	1.4 ± 2.7
ICM origin 1	52.9 ± 6.5	−47.7 ± 3.8	−56.4 ± 5.4	51.0 ± 4.4	−46.9 ± 4.7	−54.9 ± 4.4
ICM origin 4	10.3 ± 6.0	−28.2 ± 4.1	−30.1 ± 4.6	17.2 ± 9.2	−24.4 ± 5.3	−30.9 ± 4.9
ICM insertion 1	77.1 ± 7.7	−52.0 ± 7.1	−1.8 ± 2.5	81.1 ± 7.4	−51.3 ± 5.9	0.0 ± 1.6
ICM insertion 2	37.9 ± 5.4	−21.0 ± 5.2	−1.5 ± 2.5	51.8 ± 10.4	−18.7 ± 8.6	0.8 ± 1.7
COC insertion 1	82.4 ± 7.8	−59.5 ± 7.4	−5.0 ± 2.9	81.7 ± 9.5	−55.8 ± 7.4	−2.6 ± 2.0
COC insertion 2	54.9 ± 7.6	−46.2 ± 8.0	−4.8 ± 2.8	60.3 ± 10.3	−39.4 ± 9.0	−2.2 ± 2.2

*PVM* pubovisceral muscle, *PVaM* pubovaginal muscle, *PPM* puboperineal muscle, *PAM* puboanal muscle, *PRM* puborectal muscle, *ICM*iliococcygeus muscle, *COC* coccygeus muscle

### 3D Origin and Insertion Points of LAM

Origin points were similar, with the lowest SD in the second PRM origin point, showing 1.6–3.6 mm in controls and 1.9–5.6 mm in cases. The PVM origin shows a slightly more posterior (x-axis) position in cases of 5 mm on the right side and 4.4 mm on the left side. Insertion points differed more, being especially pronounced in PVaM, PPM, and PAM, which were located more posteriorly (x-axis), caudally (y-axis), and centered (z-axis) in the cases, with the highest SD for the PPM insertion on the x-axis at 22.0 ± 5.0 mm in controls and 36.6 ± 8.6 mm in cases. The PRM insertion was also posteriorly displaced (x-axis) in cases (Table 2, Appendix 1).

### Distances

**Fig. 2:**
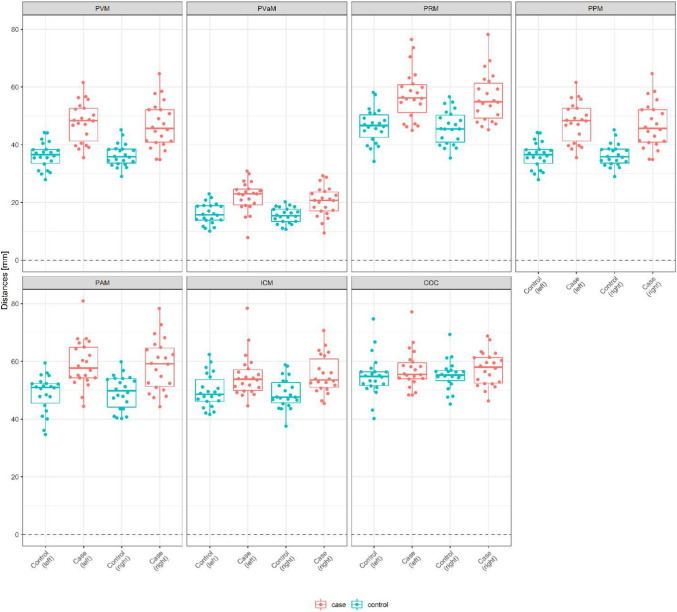
Mean distances in mm ± SD and median with first and third quartiles (interquartile range). *PVM* pubovisceral muscle, *PVaM* pubovaginal muscle, *PRM* puborectal muscle, *PPM* puboperineal muscle, *PAM* puboanal muscle, *ICM*iliococcygeus muscle, *COC* coccygeus muscle

The mean lengths were longer in cases than in controls; this was especially pronounced in PVM, PPM, PAM, and PRM, with differences of 11.5 (24%)/10.5 (22%) mm on the left/right side for the PVM, 11.5 (24%)/10.5 (22%) mm for the PPM, 10.3 (17%)/9.6 (16%) mm for the PAM, and 10.8 (19%)/10.3 (18%) mm for the PRM (Fig. 2). The COC showed the smallest difference, with 2.3 mm (4%) on the left side and 2.2 mm (4%) on the right side.

### Angles

**Fig. 3:**
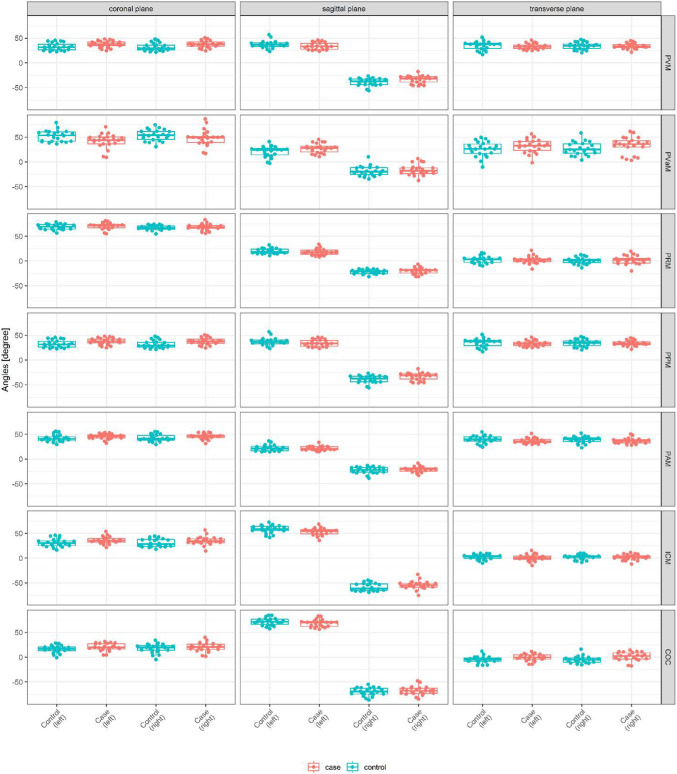
Mean angles in degrees ± SD in coronal, sagittal, and transverse planes and median with first and third quartiles (interquartile range). *PVM* pubovisceral muscle, *PVaM* pubovaginal muscle, *PRM* puborectal muscle, *PPM* puboperineal muscle, *PAM* puboanal muscle, *ICM*iliococcygeus muscle, *COC* coccygeus muscle

In the sagittal plane, angles for all muscles examined, except for the PVaM, were greater in cases (Fig. 3). In the coronal plane, angles were greater in controls, again except for the PVaM. In the axial plane, angles were greater in controls, except for the PVaM and PRM.

### Intraclass Correlation

**Fig. 4:**
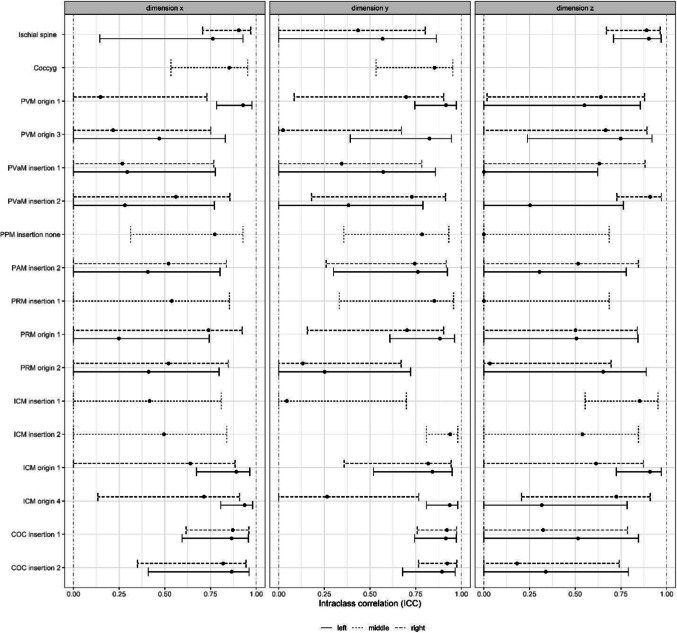
Inter-rater reliability (intra-class coefficients) with 95% confidence intervals, separately for all dimensions and body side. *Coccyg* coccygeus, *PVM* pubovisceral muscle, *PVaM* pubovaginal muscle, *PRM* puborectal muscle, *PPM* puboperineal muscle, *PAM* puboanal muscle, *ICM*iliococcygeus muscle, *COC* coccygeus muscle

Moderate to good ICC was reported for all observed muscles, with the exception of points close to point 0/0/0 (Fig. 4). However, the 95% confidence intervals are quite large and do not allow for precise statements on interrater agreement.

The ICC on the z-axis for the coccyx bone point could not be calculated because it is defined as 0. The symphysis organ point is not included because all values are defined as 0.

## Discussion

Our study provides a detailed map of the 3D coordinates for LAM subdivisions in two premenopausal collectives with and without POP. It is the first morphometric analysis of the LAM within a standardized 3D coordinate system. The study delivers exactpoint locations for the female pelvic floor muscles independent of pelvic tilt or position. This novel biometric assessment allows an in-depth 3D analysis of LAM characteristics in asymptomatic and symptomatic young women and reveals distinct differences in the spatial distribution of their muscles.

### Demographics

The cases (premenopausal, parous women with POP-Q ≥ II) were more symptomatic in all four pelvic domains. The age difference between control and case group women can be explained by the fact that nulliparity or primi-/multiparity and POP symptoms respectively were required. Comparing groups of the same age would require further studies. With a clearly premenopausal collective, substantial hormonal bias can be excluded and LAM differences are attributable to vaginal parity. With parity being an important risk factor for POP, premenopausal nulliparous women were found to have a prevalence of POP < 1% in a large-scale health survey [25]. In order to investigate purely birth-mechanical changes, groups would have to be formed of nulliparous and parous women with and without symptoms.

### 3D Morphometry

The distribution pattern with greater differences in the insertion points, which is especially pronounced in PVM subdivisions, suggests lesions in the course of or at the distal end of the insertion and fewer avulsions in the origin at the bone attachments. This confirms the results of a delivery simulation study showing that the PVM undergoes the longest stretch of all LAM subdivisions, resulting in the greatest risk for stretch-related injuries [26]. The fact that all distances are longer in cases indicates lengthened muscles.

The analysis revealed that in premenopausal women with POP, the insertions of the LAM subdivisions are mostly found in a more posterior and caudal location, whereas in asymptomatic premenopausal women the origins showed no change. This lower positioning results in muscles lengthening and organs gliding caudally and toward the center (midsagitally). Most muscles, again especially PVM and its subdivisions, showed a more posterior positioning up to 14.6 mm in the PPM insertion point in the symptomatic cases, whereas the differences in the coronal plane are overall small and show no clear pattern.

The ICC compares the variability between values with the variability between raters. The ICC value depends on the scale, meaning that if the differences between raters are the same, but the variability between subjects is smaller, the ICC tends to become smaller. This happens when the muscle points are closer to the origin of the coordinate system. The ICC values between points are therefore not directly comparable [27].

In a recently published feasibility study of LAM quantification using 3D PICS in 35 nulliparous women, the origin and insertion points were comparable overall, with small to moderate differences in the PVM origin, PVaM insertion, and COC insertion, which were all more posteriorly located in the feasibility study. Furthermore, the PRM insertion was more cranially located in the feasibility study and the ICM insertion was both more cranially and more posteriorly located in that study [16]. The ligaments of the pelvic floor were already characterized using that method, providing the basis for a better understanding of the pelvic organ support structures [28].

3D video animations of the in vivo anatomy of controls and cases in 3D PICS will represent further advances on earlier single-patient animations [29].

The limitations of this study are its small sample size, having to image participants in a supine position and the missing analysis of dynamic sequences and muscle details. Dynamic sequences were recorded, but the LAM subdivisions were no longer separately identifiable. The small sample size with little subject diversity regarding height, weight, and ethnicity plus the age gap between the groups demand confirmation in a larger collective to allow for generalization. There was no vaginal examination of the control group. Also, possible confounders such as BMI, age, and comorbidities should be the subject of further studies, as well as a comparison of symptomatic and asymptomatic parous women. There are no data on muscle size or thickness, compartment-specific defects, surrounding structures, or interrelation of supporting structures such as ligaments, arcus tendineus, or connective tissue. LAM avulsion and nerve injuries due to vaginal birth-induced strain forces on the neuromuscular tissue were not studied in detail either. Major LAM avulsion was present in 9 patients of the case group (41%), consistent with a more posterior position of the PVM origin 1 in cases. Because not all patients were rated by both raters, the ICC estimates come with some uncertainty; they are, however, comparable with the methodology study of Moser et al. [16]. There remains the possibility of worse general interrater agreement in untrained physicians.

The strengths of the study are the controlled setting with controls and patients, the multimodal assessment using a questionnaire, clinical examination, the muscle-specific MRI protocol, and the agreement analysis. In contrast to early MRI studies, where, despite standardized central training, a high variability in pelvic MRI measurements among readers–particularly for soft-tissue structures–was found [30], our ICC showed moderate to good agreement.

## Conclusion

This study is to our knowledge the first to use a quantitative approach to MRI to assess differences in LAM subdivisions between premenopausal women with symptomatic POP and asymptomatic controls. Our results showed that there were measurable differences in insertion sites, muscle length, and angles. To establish reliable threshold values for normal and pathological conditions, further and larger-scale studies, including pressure measurements, are needed. With this quantitative morphometric approach, we are able to offer a new tool in studying defect mechanisms and enabling the development of future precision diagnostics in the pathogenesis of POP.

## Electronic supplementary material

Below is the link to the electronic supplementary material.

Supplementary file1 (PDF 1877 KB)

Supplementary file2 (MP4 53986 KB)

## Data Availability

Following the open access policy of the University of Zurich and the Swiss National Research Foundation, data will be available after publication via open access on the University of Zurich Open Access Repository and Archive (ZORA), https://www.zora.uzh.ch/.

## Appendix 1

**Table 3:** Right-sided analysis of the marked 3D PICS muscle, organ and bone points (x/y/z) on each axis, mean values are expressed in mm (± SD)

Point	Controls			Cases		
	x	y	z	x	y	z
Bladder	−0.7 ± 5.3	−26.5 ± 2.1	−1.4 ± 3.5	7.4 ± 8.2	−18.5 ± 9.4	1.4 ± 3.9
Cervix	36.4 ± 12.5	−55.2 ± 7.6	−8.2 ± 10.4	37.9 ± 11.7	−45.0 ± 15.8	0.1 ± 10.0
Anorectum	27.7 ± 4.5	−23.5 ± 6.8	−1.1 ± 3.2	35.7 ± 6.9	−15.9 ± 8.5	0.7 ± 4.5
Symphysis	0.0 ± 0.0	0.0 ± 0.0	0.0 ± 0.0	0.0 ± 0.0	0.0 ± 0.0	0.0 ± 0.0
Ischial spine	55.3 ± 6.1	−47.8 ± 3.9	53.5 ± 5.2	53.4 ± 5.2	48.0 ± 4.4	56.8 ± 5.8
Coccyx	91.7 ± 6.3	−61.8 ± 4.3	0.0 ± 0.0	92.2 ± 5.8	−62.2 ± 3.9	0.0 ± 0.0
PVM origin 1	9.8 ± 4.4	−34.1 ± 3.7	27.0 ± 5.0	14.8 ± 4.4	−29.3 ± 5.7	32.9 ± 5.8
PVM origin 3	−4.1 ± 3.6	−14.0 ± 2.7	13.7 ± 4.2	1.4 ± 5.7	−12.4 ± 3.5	20.0 ± 7.2
PVaM insertion 1	5.4 ± 3.0	−12.4 ± 4.0	14.0 ± 2.8	12.3 ± 5.2	−5.0 ± 6.4	19.8 ± 4.3
PVaM insertion 2	17.9 ± 3.0	−11.7 ± 3.8	10.0 ± 2.9	26.0 ± 5.9	−5.6 ± 5.3	15.0 ± 7.4
PPM insertion	22.0 ± 5.0	−3.6 ± 5.6	−1.9 ± 2.8	36.6 ± 8.6	5.2 ± 7.2	1.1 ± 3.0
PAM insertion	39.2 ± 6.0	1.5 ± 6.7	5.0 ± 3.0	53.4 ± 10.1	9.5 ± 7.2	8.8 ± 3.0
PRM origin 1	−7.2 ± 3.0	−11.4 ± 3.0	15.5 ± 3.6	−5.3 ± 2.7	11.4 ± 3.1	20.1 ± 5.6
PRM origin 2	3.0 ± 2.1	−2.4 ± 1.3	15.4 ± 3.6	4.6 ± 2.7	−1.9 ± 1.9	20.1 ± 5.6
PRM insertion 1	40.3 ± 6.1	−6.5 ± 5.1	−1.4 ± 2.5	51.7 ± 8.7	−4.7 ± 7.8	1.4 ± 2.7
ICM origin 1	52.7 ± 6.2	−47.9 ± 3.8	53.4 ± 4.8	51.8 ± 5.4	−47.9 ± 4.3	56.1 ± 5.9
ICM origin 4	12.1 ± 5.7	−28.2 ± 3.9	26.7 ± 4.0	17.3 ± 10.1	−25.1 ± 5.5	33.1 ± 5.7
ICM insertion 1	77.1 ± 7.7	−52.0 ± 7.1	−1.8 ± 2.5	81.1 ± 7.4	−51.3 ± 5.9	0.0 ± 1.6
ICM insertion 2	37.9 ± 5.4	−21.0 ± 5.2	−1.5 ± 2.5	51.8 ± 10.4	−18.7 ± 8.6	0.8 ± 1.7
COC insertion 1	83.9 ± 8.0	−59.9 ± 8.6	1.1 ± 2.6	81.7 ± 9.3	−54.6 ± 8.5	3.4 ± 2.0
COC insertion 2	55.5 ± 7.5	−46.0 ± 8.3	1.3 ± 2.5	61.5 ± 10.2	−38.2 ± 10.2	3.8 ± 2.2

*PVM* pubovisceral muscle, *PVaM* pubovaginal muscle, *PPM* puboperineal muscle, *PAM* puboanal muscle, *PRM* puborectal muscle, *ICM* iliococcygeus muscle, *COC* coccygeus muscle

## Appendix 2

**Table 4:** Distances calculated using Euclidean metric, with mean ± SD

Muscle	Left		Right	
	Control, mean ± SD	Case, mean ± SD	Control, mean ± SD	Case, mean ± SD
ICM origin 1–insertion 1	60.8 ± 8.2	63.4 ± 6.2	60.4 ± 6.4	64.0 ± 7.3
ICM origin 1–insertion 2	63.8 ± 4.9	64.1 ± 5.7	62.8 ± 4.4	64.0 ± 6.1
ICM origin 4–insertion 1	76.7 ± 11.2	76.5 ± 12.3	75.2 ± 11.3	77.2 ± 12.4
ICM origin 4–insertion 2	41.2 ± 6.3	48.8 ± 10.8	39.7 ± 6.0	49.4 ± 10.0
PRM origin 1–insertion 1	52.3 ± 6.4	62.3 ± 9.3	51.0 ± 6.3	61.2 ± 8.4
PRM origin 2–insertion 1	41.6 ± 5.6	52.9 ± 8.4	41.6 ± 5.6	51.8 ± 8.5

*ICM* iliococcygeus muscle, *PRM* puborectal muscle
