# Supplementary material for: Morphometric Characterization of Levator Ani Subdivisions in Healthy Controls and Patients: An MRI Study Using 3D PICS
Source: Int Urogynecol J. 2025 Mar 20;36(6):1281–92. doi: 10.1007/s00192-025-06082-5 (PMC12287122; doi:10.1007/s00192-025-06082-5)
Supplement: Supplementary file 1 — Supplementary file1 (PDF 1877 KB) [file 192_2025_6082_MOESM1_ESM.pdf]

Appendix 3: Instructions for point placement

Orientation:

- Sagittal: anterior on the left side of the image

- Axial: right on the left side of the image, anterior on the upper side of the image

- Coronal: right on the left side of the image

**Point**	**Instructions**	**Illustration (3D-PICS-Screenshot)**
Organ point: **Bladder**	Sagittal plane, midpoint of internal bladder meatus	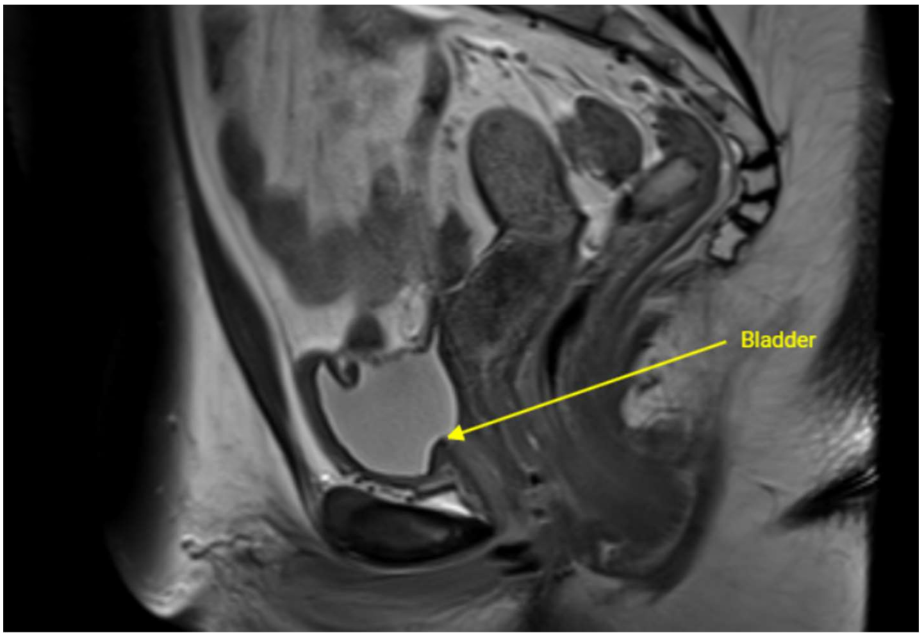
Organ point: **Cervix**	Sagittal plane, midpoint of external cervical os	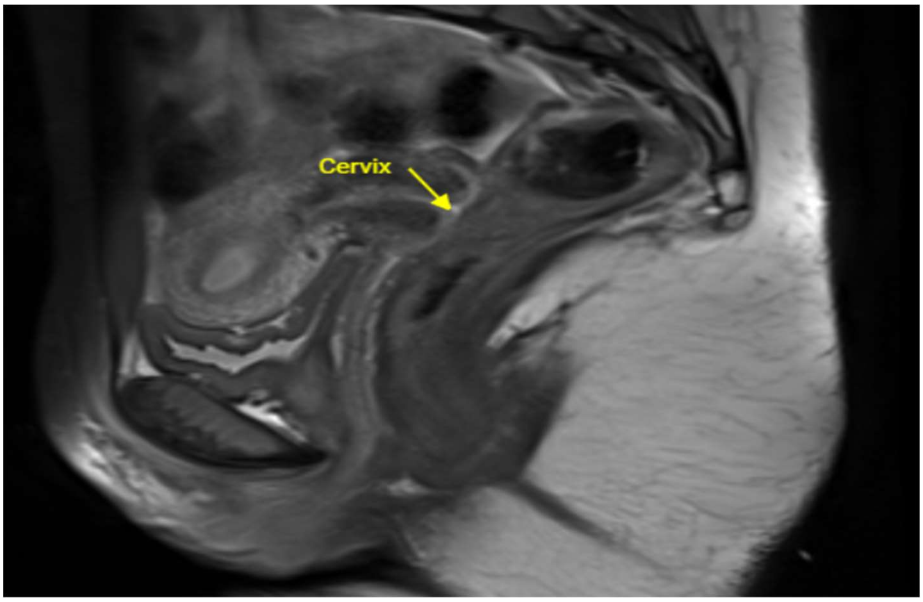

Organ point: **Anorectum**	Sagittal plane, midpoint of angulation of anus and rectum	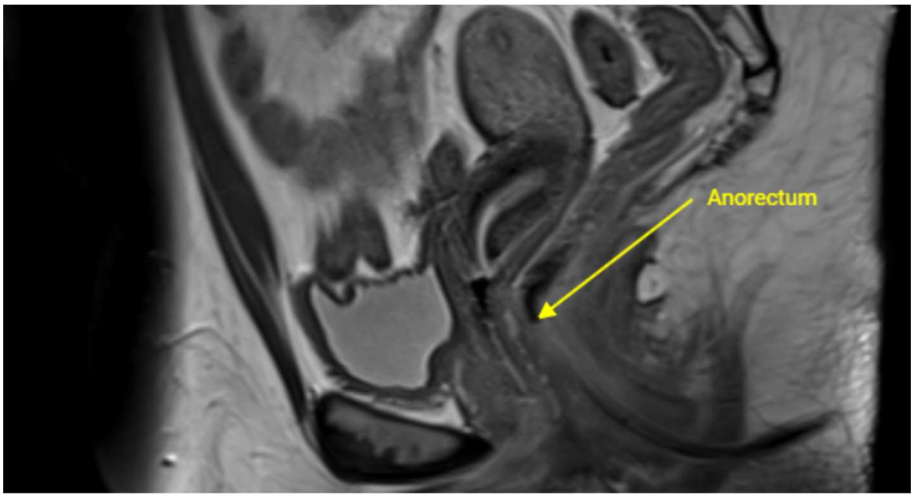
Bone point: **Symphysis**	Sagittal plane, inferior pubic point, most central	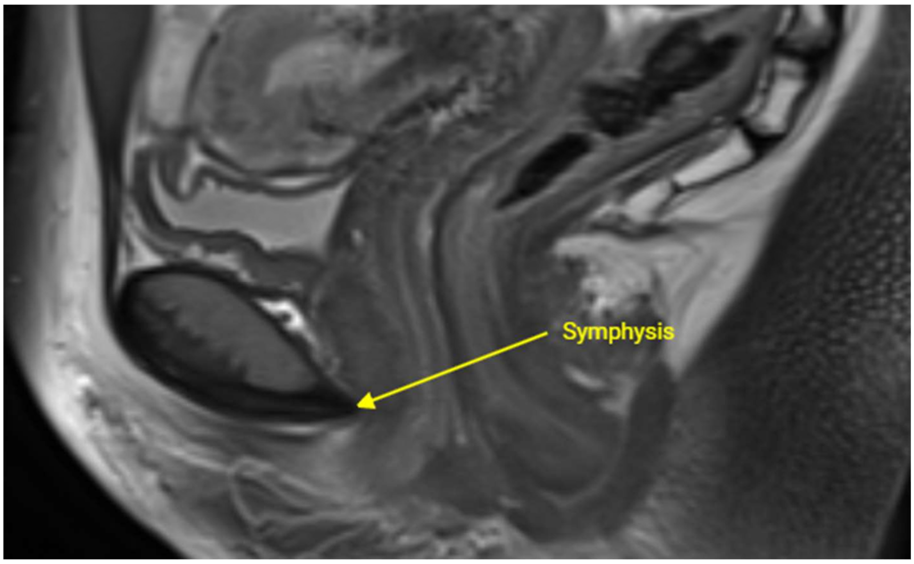
Bone point: **Ischial spine**	Axial plane, at the height of the femoral head, most anterior and medial spine point on each side	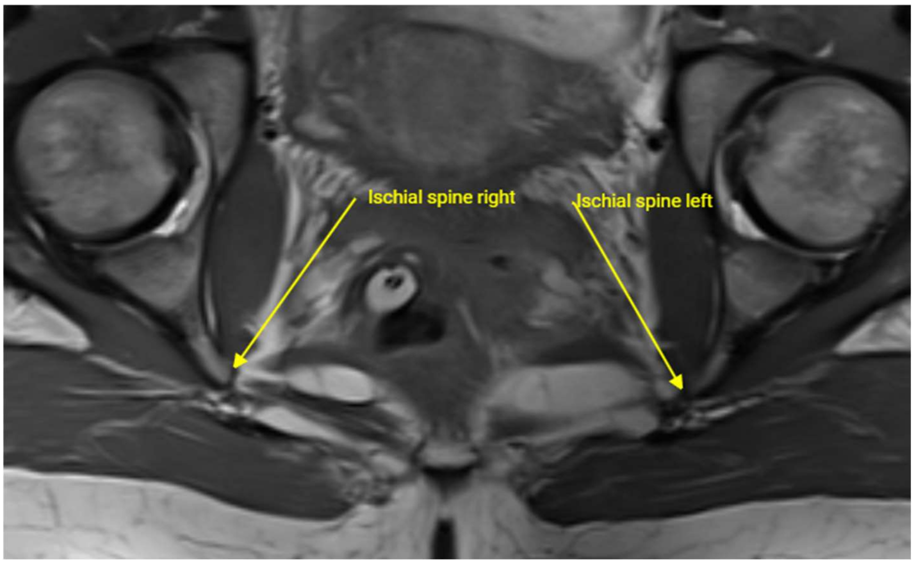

<table>
<tr>
<td>Bone point:
Coccyg</td>
<td>Sagittal plane, sacro-coccygeal joint: usually the fifth ((4th-6th), medial cranial edge of the first coccygeal bone</td>
<td>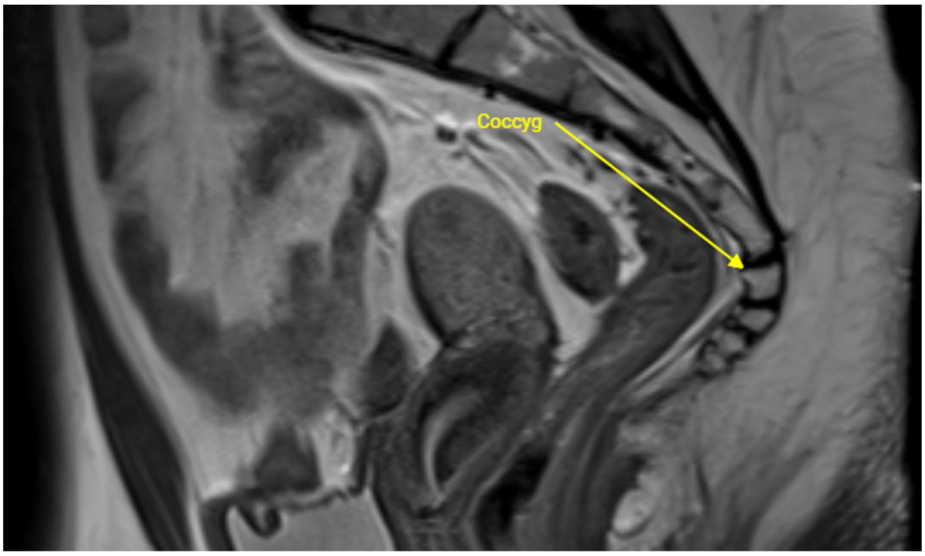
</td>
</tr>
<tr>
<td rowspan="2">Muscle point:
PVM Origin 1</td>
<td>Coronal plane, most cranial and posterior point, bird-wing-like structure</td>
<td>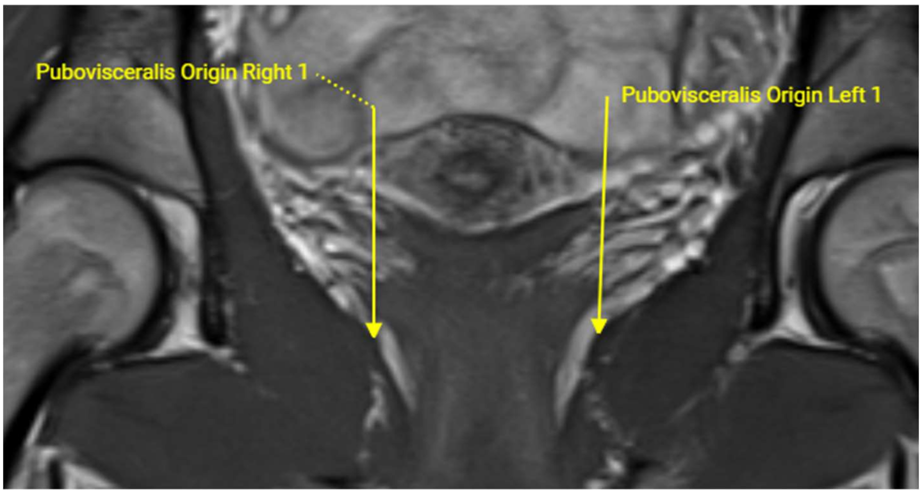
</td>
</tr>
<tr>
<td>Cross-check in axial plane, must equal ICM Origin 3</td>
<td>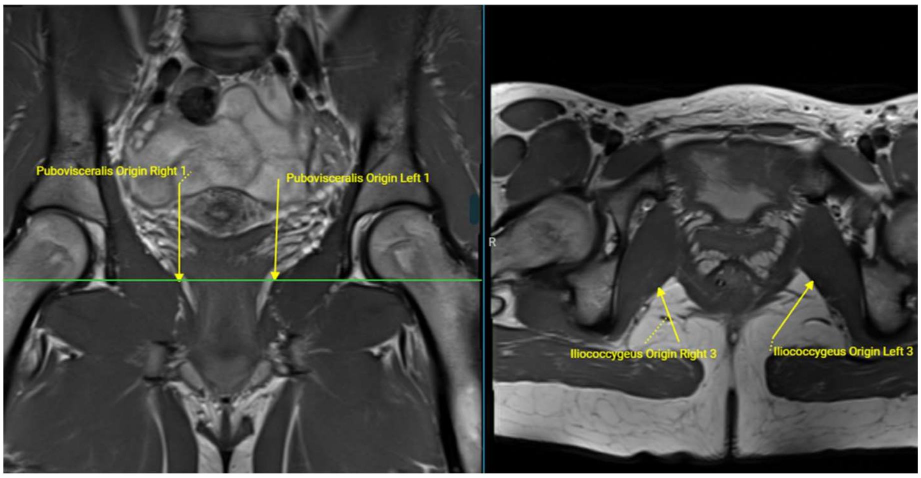
</td>
</tr>
</table>

<table>
<tr><td>Muscle point: PVM Origin 2 -not used in analysis</td><td>Coronal plane, halfway between PVM Origin and PVM Origin 3</td><td>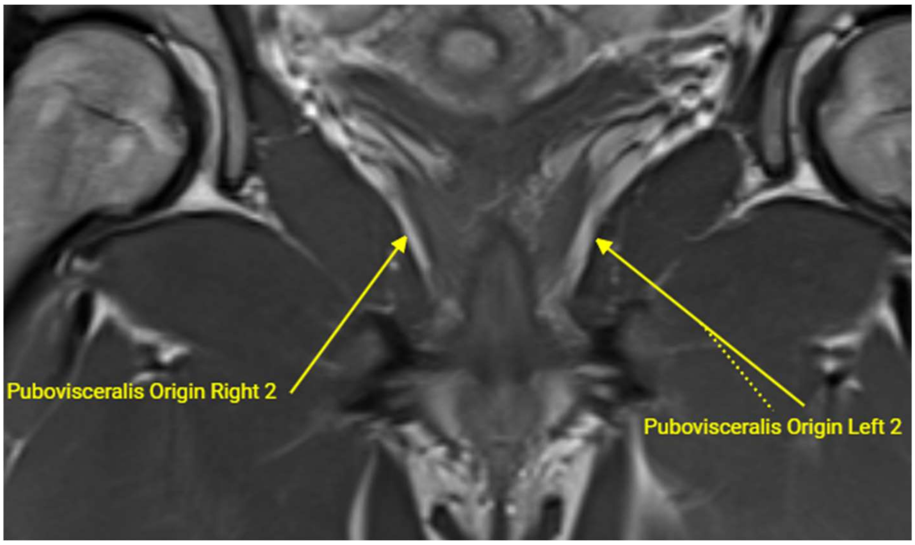
</td></tr>
<tr><td>Muscle point: PvaM Insertion 1</td><td>Axial plane, Lowest part of the vagina (“w”-shaped), most distal visible muscle fibers at anterior end of the vaginal side wall</td><td rowspan="2">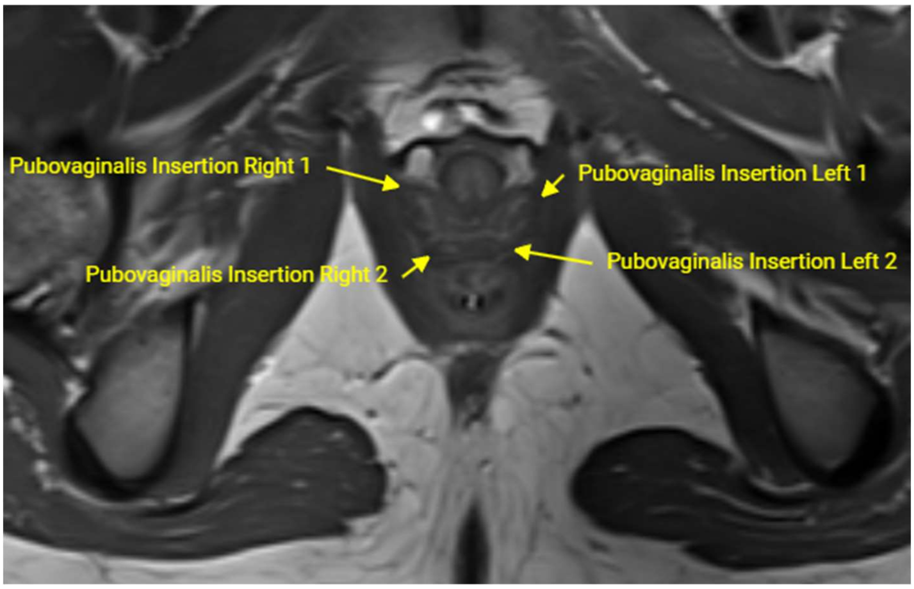
</td></tr>
<tr><td>Muscle point: PVaM Insertion 2</td><td>Axial plane, same as PvaM insertion 1, but posterior end of the vaginal side wall</td></tr>
<tr><td>Muscle point: PPM Insertion-Named “Puboperinealis Insertion Right 1” in the annotation. There was no left point, as it is a midline structure</td><td>Axial plane, lower than pubovaginal, same height or higher than puboanal, dark structure in the midline, anterior to the anal canal</td><td>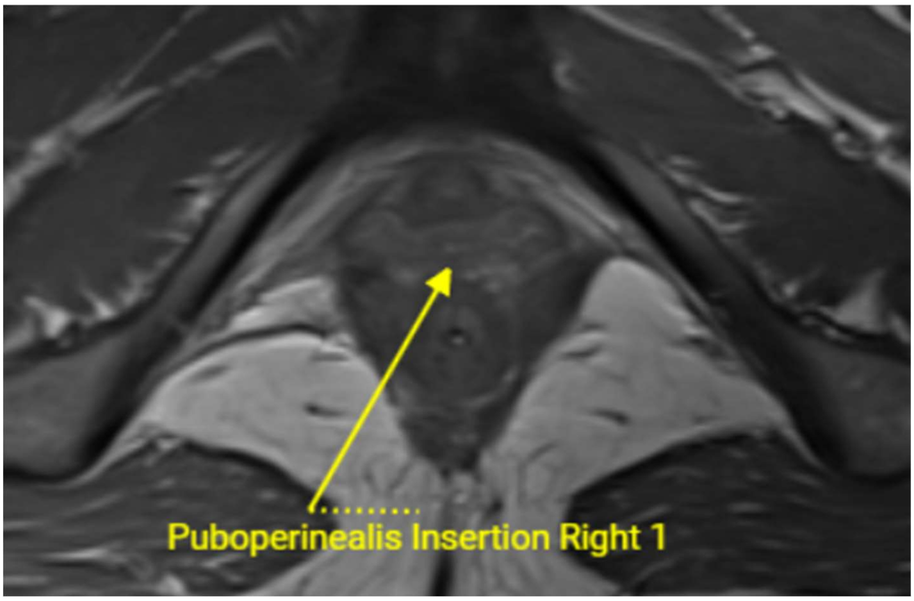
</td></tr>
</table>

<table>
<tr><td>Muscle point:
PAM Insertion 1
-not used in analysis</td><td rowspan="3">Axial plane, at the intersphincteric groove, most distal slide, PAM insertion between the most anterior (PAM insertion 1) and the most posterior (PAM insertion 3) border of the anal canal</td><td rowspan="3">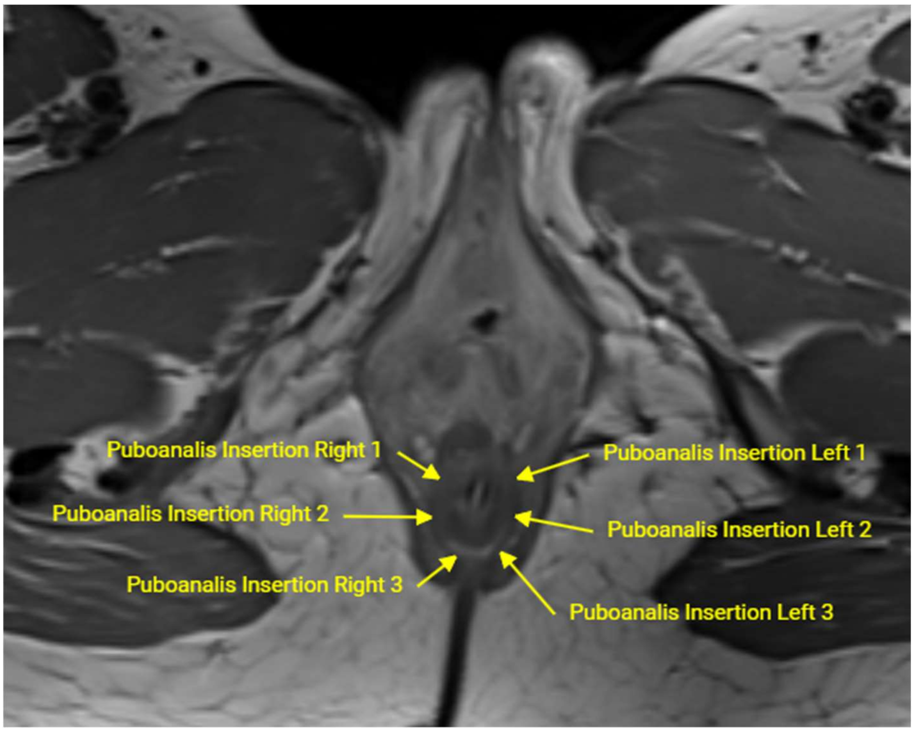
</td></tr>
<tr><td>Muscle point:
PAM Insertion
-initially named "Puboanalis Insertion 2"</td></tr>
<tr><td>Muscle point:
PAM Insertion 3
-not used in analysis</td></tr>
<tr><td>Muscle point:
PRM Origin 1
-left side shown</td><td rowspan="2">Sagittal plane, parasagittal left and right, black muscular structure adjacent to the symphysis. PRM origin 1: cranial muscle fibers, found at about one third of the length of the symphysis. PRM origin 2: muscle fibers at the inferior and posterior ends of the symphysis</td><td rowspan="2">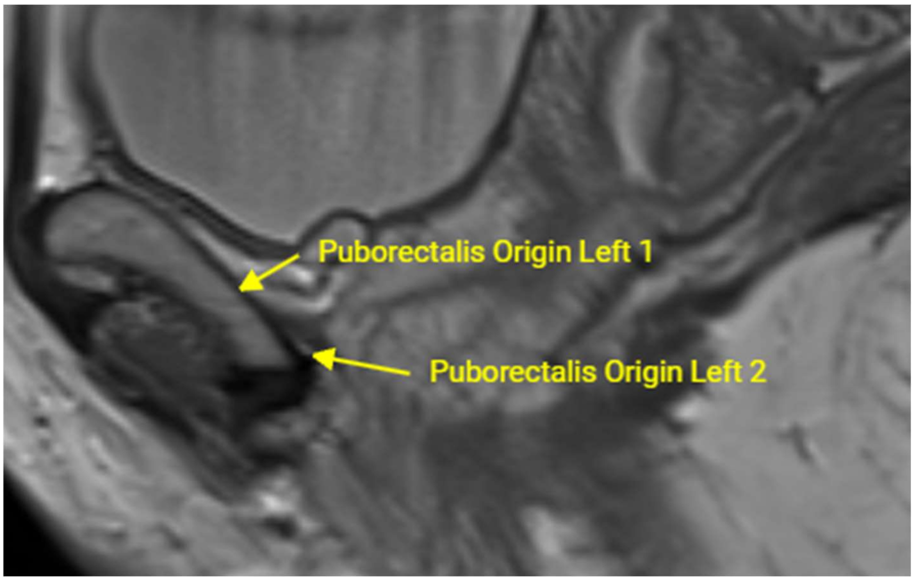
</td></tr>
<tr><td>Muscle point:
PRM Origin 2
-left side shown</td></tr>
</table>

Muscle point: **PRM Insertion 1**	Sagittal plane, midsagittal, bump behind anorectal junction. Most distal and posterior point of the bump.	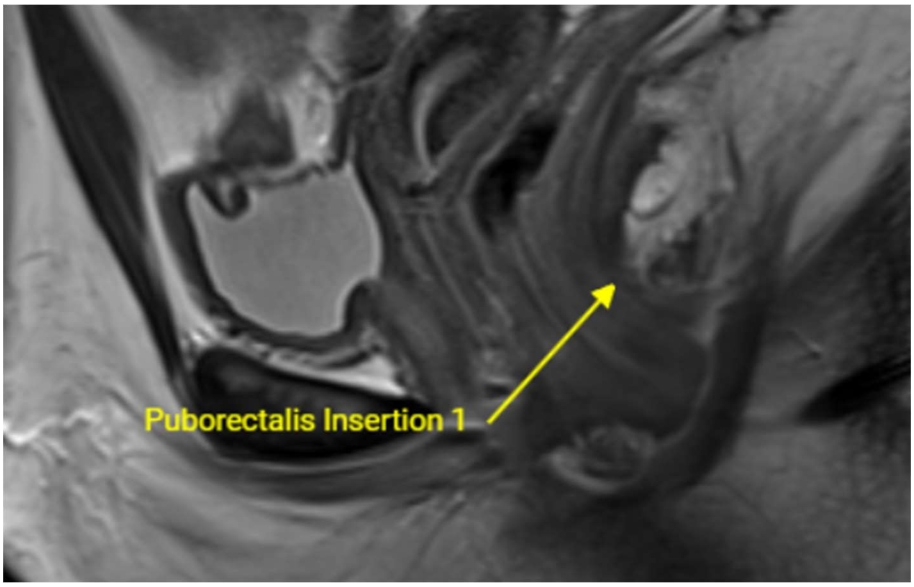
Muscle point: **ICM Origin 1**	Axial plane, ischial spine. Muscle fibers running anteriorly from the ischial spine.	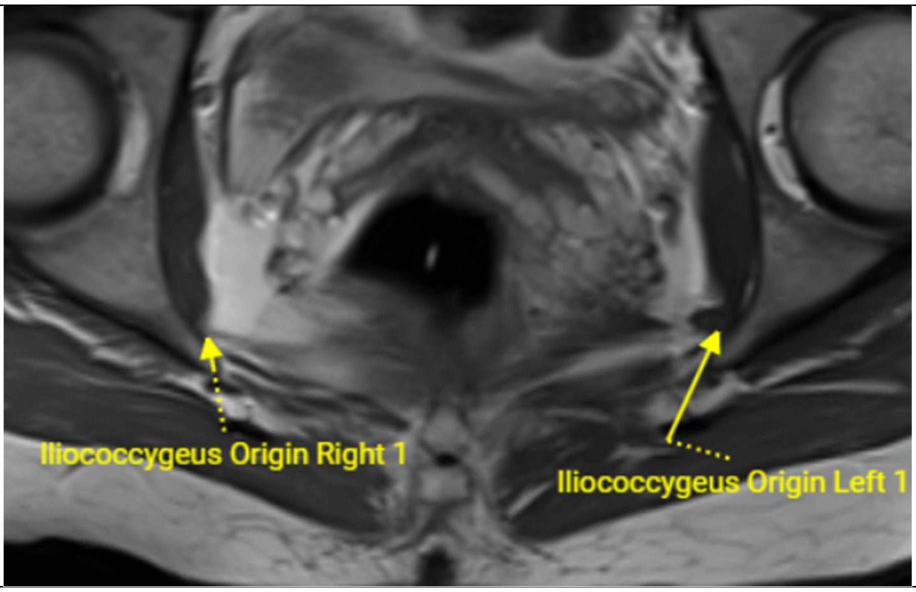
Muscle point: **ICM Origin 2** -not used in analysis	Axial plane, one third of the way between ICM origin 1 and ICM origin 4	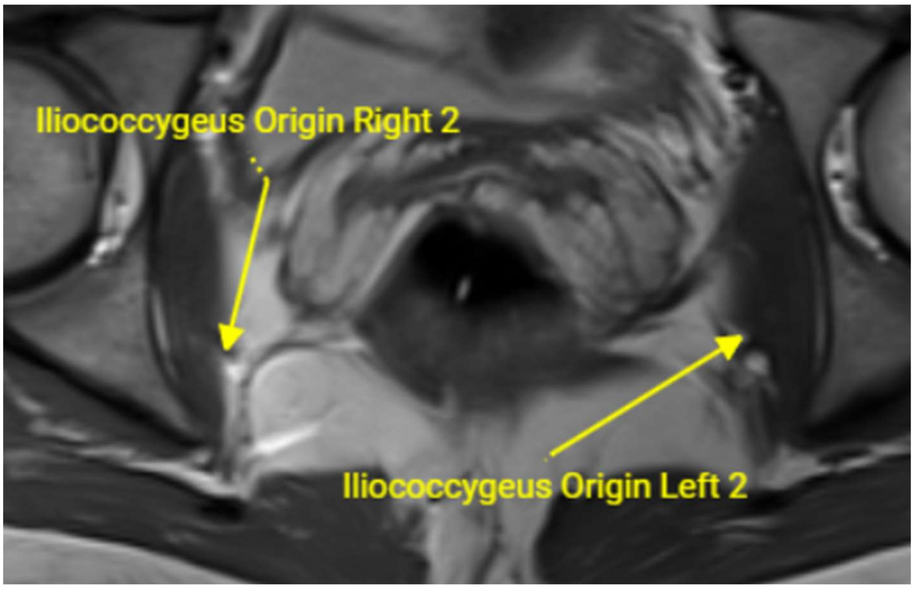

<table>
<tr>
<td>Muscle point:
ICM Origin 3
-not used in analysis</td>
<td>Axial plane, two thirds of the way between ICM origin 1 and ICM origin 4</td>
<td>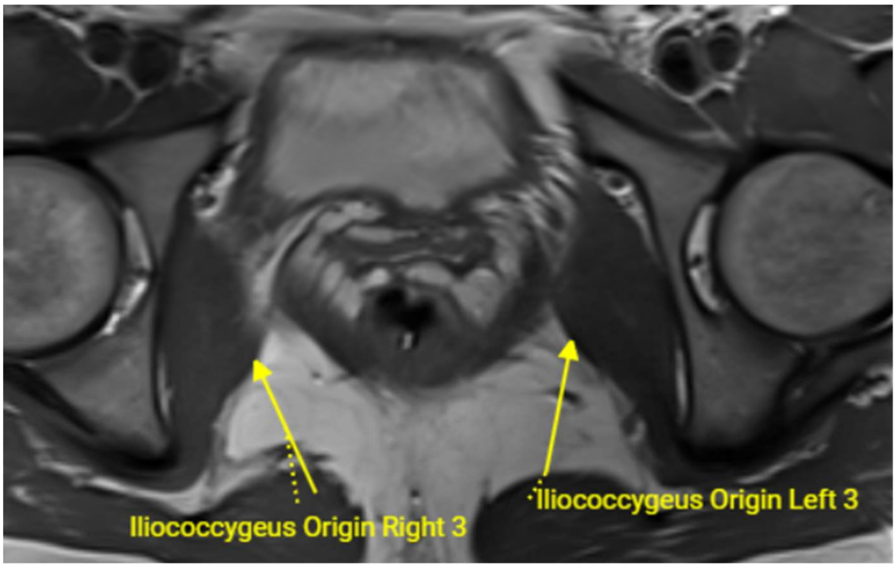
</td>
</tr>
<tr>
<td>Muscle point:
ICM Origin 4</td>
<td>Axial plane, marking the transition of the ICM to the obturator muscle, vector change of pubovisceral muscle</td>
<td>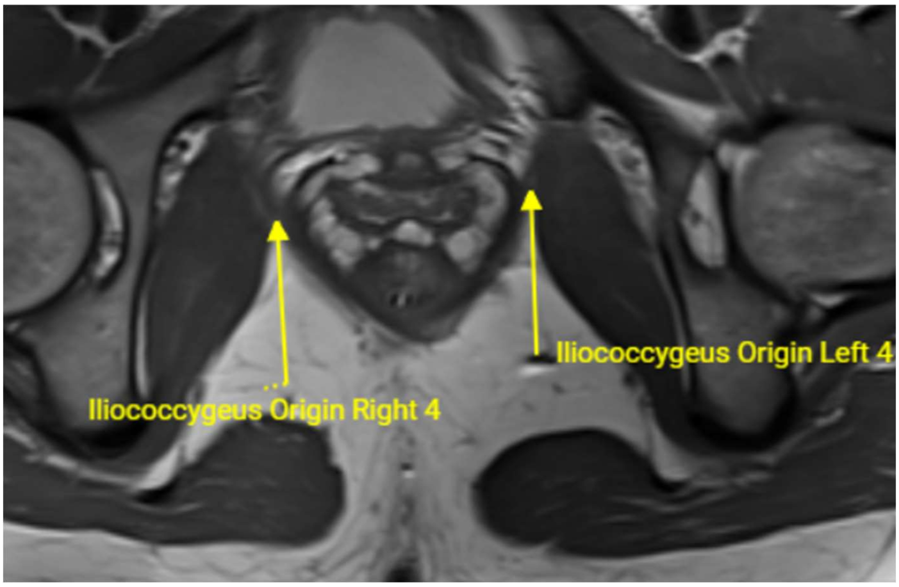
</td>
</tr>
<tr>
<td>Muscle point:
ICM Insertion 1</td>
<td rowspan="2">Sagittal plane, most cranial (ICM insertion 1) and most distal (ICM insertion 2) point along the Iliococcygeal raphe</td>
<td rowspan="2">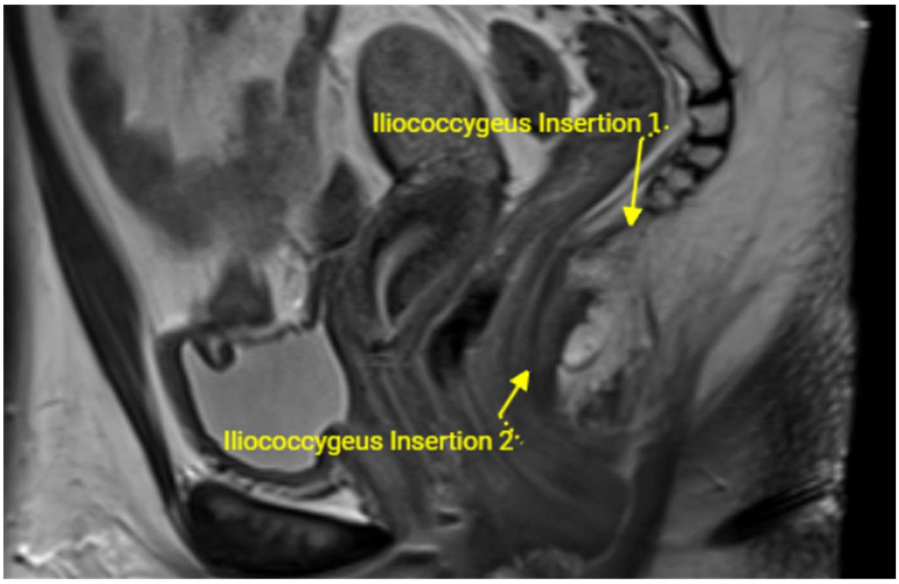
</td>
</tr>
<tr>
<td>Muscle point:
ICM Insertion 2</td>
</tr>
</table>

<table>
<tr>
<td>Muscle point:
COC Insertion 1
-right side shown</td>
<td rowspan="2">Sagittal plane, parasagittal, about 5 mm from the bone, most cranial (COC insertion 1) and most caudal (COC insertion 2) point of the thin black structure</td>
<td rowspan="2">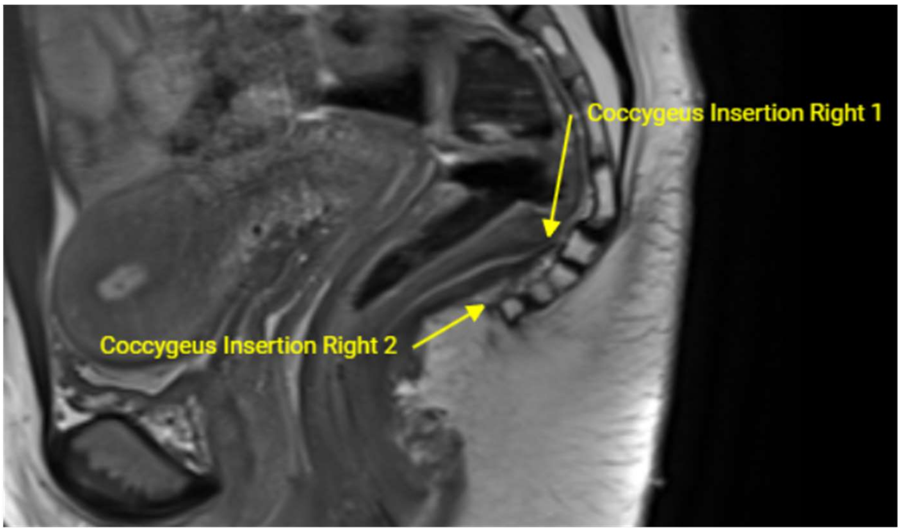
</td>
</tr>
<tr>
<td>Muscle point:
COC Insertion 2
-right side shown</td>
</tr>
</table>
